# Glucose-level dependent brain hypometabolism in type 2 diabetes mellitus and obesity

**DOI:** 10.1186/s41824-021-00097-z

**Published:** 2021-02-17

**Authors:** Z. Képes, Cs. Aranyi, A. Forgács, F. Nagy, K. Kukuts, Zs. Hascsi, R. Esze, S. Somodi, M. Káplár, J. Varga, M. Emri, I. Garai

**Affiliations:** 1 Faculty of Medicine, Department of Medical Imaging, Division of Nuclear Medicine and Translational Imaging, University of Debrecen, Nagyerdei krt. 98, Debrecen, Hungary; 2 Scanomed Ltd. Nuclear Medicine Centers, Nagyerdei krt. 98, Debrecen, Hungary; 3 Faculty of Medicine, Department of Internal Medicine, University of Debrecen, Nagyerdei krt. 98, Debrecen, Hungary

**Keywords:** [^18^F]FDG, Metabolism, Brain, Type 2 diabetes mellitus, Obesity

## Abstract

**Background:**

Metabolic syndrome and its individual components lead to wide-ranging consequences, many of which affect the central nervous system. In this study, we compared the [^18^F]FDG regional brain metabolic pattern of participants with type 2 diabetes mellitus (T2DM) and non-DM obese individuals.

**Methods:**

In our prospective study, 51 patients with controlled T2DM (ages 50.6 ± 8.0 years) and 45 non-DM obese participants (ages 52.0 ± 9.6 years) were enrolled. Glucose levels measured before PET/CT examination (pre-PET glucose) as well as laboratory parameters assessing glucose and lipid status were determined. NeuroQ application (NeuroQ^TM^ 3.6, Syntermed, Philips) was used to evaluate regional brain metabolic differences. [^18^F]FDG PET/CT (AnyScan PC, Mediso) scans, estimating brain metabolism, were transformed to MNI152 brain map after T1 registration and used for SPM-based group comparison of brain metabolism corrected for pre-PET glucose, and correlation analysis with laboratory parameters.

**Results:**

NeuroQ analysis did not reveal significant regional metabolic defects in either group. Voxel-based group comparison revealed significantly (*P*_FWE_<0.05) decreased metabolism in the region of the precuneus and in the right superior frontal gyrus (rSFG) in the diabetic group as compared to the obese patients. Data analysis corrected for pre-PET glucose level showed a hypometabolic difference only in the rSFG in T2DM. Voxel-based correlation analysis showed significant negative correlation of the metabolism in the following brain regions with pre-PET glucose in diabetes: precuneus, left posterior orbital gyrus, right calcarine cortex and right orbital part of inferior frontal gyrus; whilst in the obese group only the right rolandic (pericentral) operculum proved to be sensitive to pre-PET glucose level.

**Conclusions:**

To our knowledge, this is the first study to perform pre-PET glucose level corrected comparative analysis of brain metabolism in T2DM and obesity. We also examined the pre-PET glucose level dependency of regional cerebral metabolism in the two groups separately. Large-scale future studies are warranted to perform further correlation analysis with the aim of determining the effects of metabolic disturbances on brain metabolism.

## Introduction

Metabolic diseases, such as type 2 diabetes mellitus (T2DM) and obesity, represent a major and escalating public-health problem worldwide. Due to wide-spread lifestyle changes, lack of physical activity, high fat and refined carbohydrate rich diet, and further environmental, genetic and epigenetic factors, the incidence and the prevalence of metabolic diseases are gaining traction. Both T2DM and obesity are clinically important components of today’s endemic commonly referred to as metabolic syndrome (MetS) (Ginsberg & MacCallum, 2009). In general, more than 1 billion people have met the criteria of MetS worldwide (Saklayen, 2018). The prevalence of MetS is associated with that of obesity (Rochlani et al., 2017). According to a WHO report, between the 1980s and 2013, there has been a 27.5% increase in the number of obese adults whilst the number of overweight children has risen by 47.1% (Ng et al., 2014). Obesity is considered to be responsible for 3.4 million deaths annually (Kelishadi, 2014). Besides the development of musculoskeletal, vascular and malignant diseases, obesity may ultimately lead to the development of T2DM (Bray, 2004). Based on recent data, the prevalence and incidence of T2DM also continue to increase: in 2017, the global incidence and prevalence were 22.9 million and 476.0 million, whilst by 2025 these are estimated to reach 26.6 million and 570.9 million, respectively (Bhupathiraju & Hu, 2016; Lin et al., 2020).

The components of MetS lead to wide-ranging consequences, many of which affect the central nervous system. Rising evidence suggests that metabolic diseases lead to significant cerebral microvascular impairments; however, the underlying mechanism is not yet fully understood. Since MetS has a staggering global prevalence and concomitantly deteriorating quality of life as well as reduced life expectancy, there is an increasing interest in understanding the pathological mechanisms underlying the association between MetS and its effect on brain function (Yates et al., 2012; Etchegoyen et al., 2018). Understanding the molecular basis of the relationship between MetS and its impact on cerebral function may provide opportunity to discover new targets for therapeutic intervention and drug development which could be useful tools in both the prevention and deceleration of the progression of metabolic disturbances.

Several pathophysiological factors associated with obesity and T2DM could possibly be responsible for the occurrence of microcircular brain changes. Fluctuating glucose levels, insulin resistance and related altered insulin signalling together with oxidative stress may lead to neuroinflammation, which can eventually contribute to the appearance of MetS-associated brain abnormalities (Alfaro et al., 2018; Kordestani-Moghadam et al., 2020).

As we are facing an increasing incidence of MetS, the effects of metabolic diseases on the central nervous system are becoming frequently investigated. Based on existing research findings, MetS is associated with the development of subclinical neurological alterations. In an MR study carried out by Hirokazu B. et al., an increased prevalence of silent brain infarctions, periventricular hyperintensity and subcortical white matter lesions were experienced in connection with MetS (Bokura et al., 2008). Further, Segura et al. found microstructural white matter impairments in the frontal lobes of patients with MetS (Segura et al., 2009).

PET imaging is considered to be a sensitive tool to investigate brain metabolic changes. Hyun-Yeol et al. reported concurrent low brain and high liver FDG uptake associated with MetS (Nam et al., 2017). In another study, mild hyperglycaemia was detected to induce decreased FDG uptake in the grey matter, mainly in the frontal, temporal and parietal association cortices, the posterior cingulate and in the precuneus (Kawasaki et al., 2008). Liu and co-workers observed lower glucose uptake in the brain of mice fed a high-fat diet for about 2 months, relative to controls (Liu et al., 2017). Wang and co-authors detected that obese individuals compared to lean people expressed higher fasting metabolism in the parietal somatosensory cortex regions that are responsible for the sensation of the mouth, lips and the tongue (Wang et al., 2002). In one study, frontal glucose metabolism was negatively associated with body mass index (BMI) (Volkow et al., 2009). Taken together, these subclinical alterations in cerebral metabolism and cerebrovascular reactivity may represent early brain deterioration associated with peripheral metabolic disturbances. Based on the scope of the existing literature, there is no definitive consensus so far regarding the effects of either T2DM or obesity on cerebral metabolism.

In this study, we investigated and compared the regional brain metabolic pattern with [^18^F]FDG in patients with T2DM and obesity to evaluate what cerebral metabolic alterations might be induced by metabolic disturbances as well as the association with different laboratory parameters.

## Materials and methods

### Study participants

Fifty-one patients with controlled T2DM and forty-five non-DM obese individuals were enrolled in this prospective study. Subjects were recruited from the Department of Internal Medicine of the University of Debrecen as well as from a private general medical praxis (Miskolc, Hungary). Patients were selected based on the following inclusion criteria: age between 18 and 70, manifest obesity (BMI >30 kg/m^2^) or controlled T2DM, and no history of mental or brain disorders. Exclusion criteria involved gravidity, breastfeeding, acute or chronic inflammatory disease, severe liver disease, ongoing steroid treatment, hyperthyroidism, retinoid intake, history of malignancies, crural ulcer, changes in therapy in the previous 6 months, use of anticoagulant treatment, and brain injury or cerebrovascular event in medical history. Before enrolment, subjects were provided with detailed pieces of information concerning the aims of the study as well as the examinations. Informed consent was collected from all patients involved (OGYEI/2829-4/2017).

### [^18^F]FDG PET/CT

To investigate cerebral metabolism, all participants underwent brain [^18^F]FDG-PET/CT examinations applying AnyScan PET/CT (Mediso, Hungary). PET acquisition was initiated 45 min (+/− 5 min) after injecting 3.5 MBq/Bw [^18^F]FDG intravenously using an automated infusion system (MEDRAD Intego, Bayer). A low-dose CT was also performed for attenuation correction. The parameters of static PET acquisition were the following: 10 min/FOV, with voxel size of 2×2×2 mm and matrix size of 160×160×76, whilst low-dose CT parameters were as follows: 120 kVp and 100 mAs. In case of 48 type 2 diabetic and 30 non-DM obese patients, additional T1-weighted 3D MR (Achieva 3.0T (TX)-DS, Philips) images were carried out for brain mapping with a voxel size of 0.5×0.5×1 mm and matrix size of 480×480×175. Before PET imaging, the patients’ actual glucose level was measured (pre-PET glucose). Patients with pre-PET glucose >12 mmol/L were rescheduled after glucose control.

### Laboratory assays

Besides serum glucose and HbA1c levels, the following laboratory parameters were also determined: sensitive thyroid-stimulating hormone, triglyceride, cholesterol, high-density lipoprotein cholesterol, low-density lipoprotein cholesterol, apolipoprotein A-I, apolipoprotein B. Pre-PET glucose level was estimated applying a test strip from finger capillary sample right before the start of the PET/CT examination (ACCU-CHEK® *Performa,* Roche Diagnostics).

### Image processing and assessment of regional brain metabolism

Two separate evaluation paths were followed.

### Assessment of regional brain metabolism with NeuroQ software

FDA-approved NeuroQ application (NeuroQ^TM^ 3.6, Syntermed, Philips) was used to analyse regional brain metabolism of each study participant and to statistically compare the individual’s scan with an age and sex matched normal whole brain atlas (Bridges et al., 2020). Participants’ [^18^F]FDG PET files were imported in DICOM format into the NeuroQ software programme for automatic quantification, and the activity in 240 standardised regions of interest (ROIs) was calculated. Those regions were considered abnormal which had the uptake values below 1.65 SD of the mean of the normal database.

### Statistical parametric mapping (SPM) analysis for group comparison and correlation analysis

For SPM analysis, image preprocessing was performed by an in-house developed pipeline, which involved four main procedures. Firstly, T1 weighted images of 71 patients were transformed to the MNI152 template using the ANTS linear and non-linear image registration software, with a 2×2×2 mm voxel size (Avants et al., 2008). Then, the PET images of these subjects were registered to their transformed T1 with the FLIRT linear registration tool (Jenkinson & Smith, 2001). We used these spatially normalised images to create an FDG template specific to our population by averaging the transformed PET images. Thereafter, we applied linear transformation on every subject’s FDG-PET image to create a study-specific FDG template. This template was used for spatial normalisation of patients’ FDG images by using the FNIRT software of FSL package (Jenkinson et al., 2012). Before the statistical model fitting, we applied the standard “Proportional thresholding SPM protocol” which set the threshold level at the 80% of the mean voxel values. Additionally, the voxel intensity values were linearly scaled to set the mean intensity of the whole brain PET images to 50. Finally, the normalised images were smoothed with a Gaussian kernel (FWHM=16 mm).

We used the SPM methods both for group comparison (two-sample *t* test) and correlation analysis (Ashburner, 2012). Statistical images were FWE-corrected (*P*<0.05) and we only considered clusters with an extent of at least 40 voxels.

## Results

Laboratory parameters, the main anthropometric characteristics of the patients and the performed diagnostic examinations are shown in Tables 1, 2, 3, 4, and 5.

**Table 1:** Laboratory parameters (following Gaussian distribution) assessing metabolic status of type 2 diabetic and non-DM obese study participants

	Type 2 diabetic participants	non-DM, obese participants		
Parameters	Mean; SD	Mean; SD	Reference value	***P*** value
**HbA1c (%)**	7.59; 1.27	5.49; 0.33	4.2-6.1%	<0.001**
**High-density lipoprotein cholesterol (mmol/L)**	1.21; 0.34	1.39; 0.32	Men: >1 mmol/L, women >1.3 mmol/L	0.013*
**Low-density lipoprotein cholesterol (mmol/L)**	3.05; 0.95	3.82; 0.93	<3.4 mmol/L	<0.001**

*P* values for parameters are derived from two-sample Student’s *t* test of the type 2 diabetic and obese group of patients; *DM* diabetes mellitus; *HbA1c* glycated haemoglobin; *SD* standard deviation

*Significance level <0.05

**Significance level <0.001

**Table 2:** Laboratory parameters (following non-Gaussian distribution) assessing metabolic status of type 2 diabetic and non-DM obese study participants

	Type 2 diabetic participants	Non-DM, obese participants		
Parameters	Median; IQR	Median; IQR	Reference value	***P*** value
**Pre-PET glucose (mmol/L)**	7.20; 2.55	6.00; 0.78	3.6-6.0 mmol/L	<0.001**
**Serum glucose (mmol/L)**	8.45; 4.48	5.40; 0.75	3.6-6.0 mmol/L	<0.001**
**Sensitive thyroid-stimulating hormone (mU/L)**	1.87; 1.32	2.21; 1.54	0.3-4.2 mU/L	0.278
**Triglyceride (mmol/L)**	1.70; 2.20	1.40; 1.00	<1.7 mmol/L	0.015*
**Cholesterol (mmol/L)**	4.90; 1.20	5.40; 1.25	<5.2 mmol/L	0.005*
**Apolipoprotein A-I (g/L)**	1.55; 0.33	1.68; 0.39	>1.15 g/L	0.175
**Apolipoprotein B (g/L)**	1.11; 0.29	1.21; 0.25	<1.0 g/L	0.045*

P values for parameters are derived from two-sample Student’s t test of the type 2 diabetic and obese group of patients; DM diabetes mellitus; IQR interquartile range; pre-PET glucose glucose level measured prior to PET/CT (hybrid positron emission tomography and X-ray computer tomography) examination; PET positron emission tomography

*Significance level <0.05

**Significance level <0.001

**Table 3:** Main anthropometric characteristics (following Gaussian distribution) of type 2 diabetic participants and non-DM obese individuals

Parameters			
	Type 2 diabetic subjects	Non-DM, obese subjects	
	Mean; SD	Mean; SD	*P* value
**Weight (kg)**	98.10; 19.45	111.89; 23.61	0.003
**Height (cm)**	170.24; 8.78	170.89; 11.44	0.897

*P* values for parameters are derived from two-sample Student’s *t* test of the type 2 diabetic and obese group of patients; *DM* diabetes mellitus; *SD* standard deviation

*Significance level <0.05*

**Table 4:** Main anthropometric characteristics (following non-Gaussian distribution) of type 2 diabetic participants and non-DM obese individuals

Parameters			
	Type 2 diabetic subjects	Non-DM, obese subjects	
	Median; IQR	Median; IQR	*P* value
**Age (year)**	50.50; 12.00	52.00; 14.50	0.424*
**BMI (kg/m**^**2**^**)**	32.81; 8.00	36.33;7.28	<0.001**

*P* values for parameters are derived from two-sample Student’s *t* test of the type 2 diabetic and obese group of patients; *DM* diabetes mellitus; *IQR* interquartile range; *BMI* body mass index

*Significance level <0.05

**Significance level <0.001

**Table 5:** Number of performed examinations on type 2 diabetic and non-DM obese participants

	Type 2 diabetic participants	Non-DM obese participants
**PET/CT**	51	45
**MRI**	48	30
**Laboratory analyses**	50	45

*DM* diabetes mellitus; *PET/CT* hybrid positron emission tomography and X-ray computer tomography; *MRI* magnetic resonance imaging

There were no significant differences in age (*t* test, *P*=0.42) and gender (chi-square test, *P*=0.9) amongst the patients involved in both groups. Serum glucose, pre-PET glucose and HbA1C levels of the patients were measured. Obviously, these parameters were significantly higher (*P*<0.001) in the diabetic group due to the presence of the disease itself. Body weight and BMI were statistically higher in the non-DM obese group which is the consequence of obesity. Comparing the two groups, in the diabetic group, higher triglyceride levels (*P*=0.01) were measured, whilst cholesterol (*P*=0.01), low-density lipoprotein cholesterol (*P*<0.001) and apolipoprotein B (*P*=0.04) levels were significantly higher in the non-DM obese group. In type 2 diabetics, high-density lipoprotein cholesterol levels were significantly lower compared to the obese (*P*=0.01). These differences could be due to the diseases themselves.

### Regional analysis of brain metabolism

**Fig. 1:**
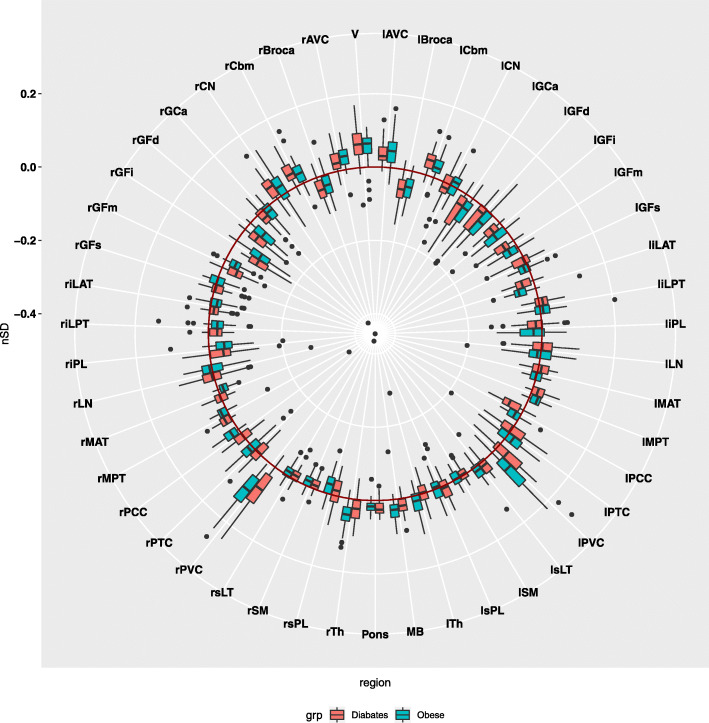
The result of NeuroQ analysis. No significant difference from normal was detected either in the type 2 diabetic or in the obese group regarding [^18^F]FDG brain uptake. However, some brain regions were depicted with lower cerebral activity but this was not considered statistically significant either. Amongst these areas, the following could be mentioned: visual cortex (V) left associative visual cortex (lAVC), left primary visual cortex (lPVC), right primary visual cortex (rPVC), right cerebellum (rCbm)

NeuroQ analysis revealed no statistically significant regional metabolic defect in either group (as demonstrated in Fig. 1). However, some brain regions were represented with lower cerebral activity, although this was not considered statistically significant either. Amongst these areas, the following could be mentioned: visual cortex (V) left associative visual cortex (lAVC), left primary visual cortex (lPVC), right primary visual cortex (rPVC), right cerebellum (rCbm).

### SPM analysis for group comparison and correlation analysis

**Fig. 2:**
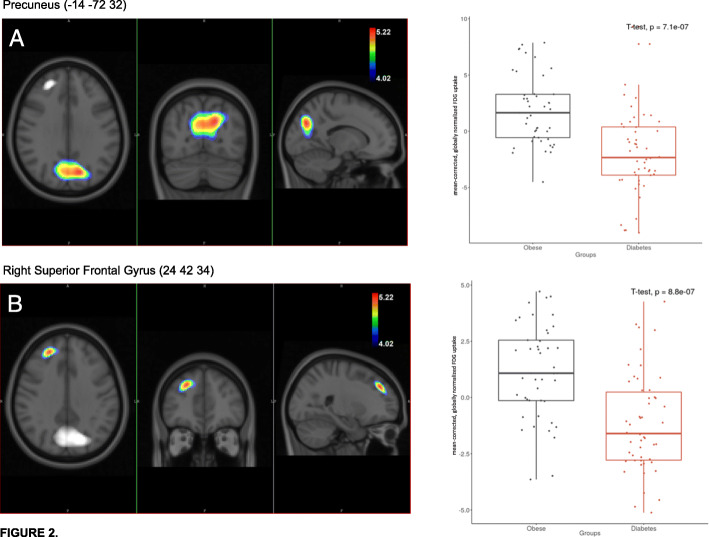
The left side show the thresholded voxel-wise SPM{t} maps overlaid on the population-averaged T1-weighted images, representing the lower [^18^F]FDG uptake in diabetes group relative to the obese group. The colour scales demonstrate the *t* values above the applied *P*_FWE_<0.05 statistical threshold. In this analysis, we did not apply correction for pre-PET blood glucose level. In the right side, the boxplots demonstrate the group difference in globally normalised FDG uptake at the highest local Student *t* maxima

Voxel-based comparison of the two groups revealed significantly decreased metabolism in the region of the precuneus and in the right superior frontal gyrus (rSFG) in the type 2 diabetic group as shown in Fig. 2 (*P*_FWE_<0.05).

Since the pre-PET glucose level of diabetic patients were significantly higher (*P*<0.05) than those of the obese, additional glucose correction was performed during SPM analysis. After pre-PET glucose correction, only the rSFG region showed hypometabolism in the diabetic group compared to the obese. Additionally, the metabolism in the region of the precuneus was detected to be inversely proportional to pre-PET glucose level, that is, the higher the pre-PET glucose level, the greater the degree of metabolic reduction was.

**Fig. 3:**
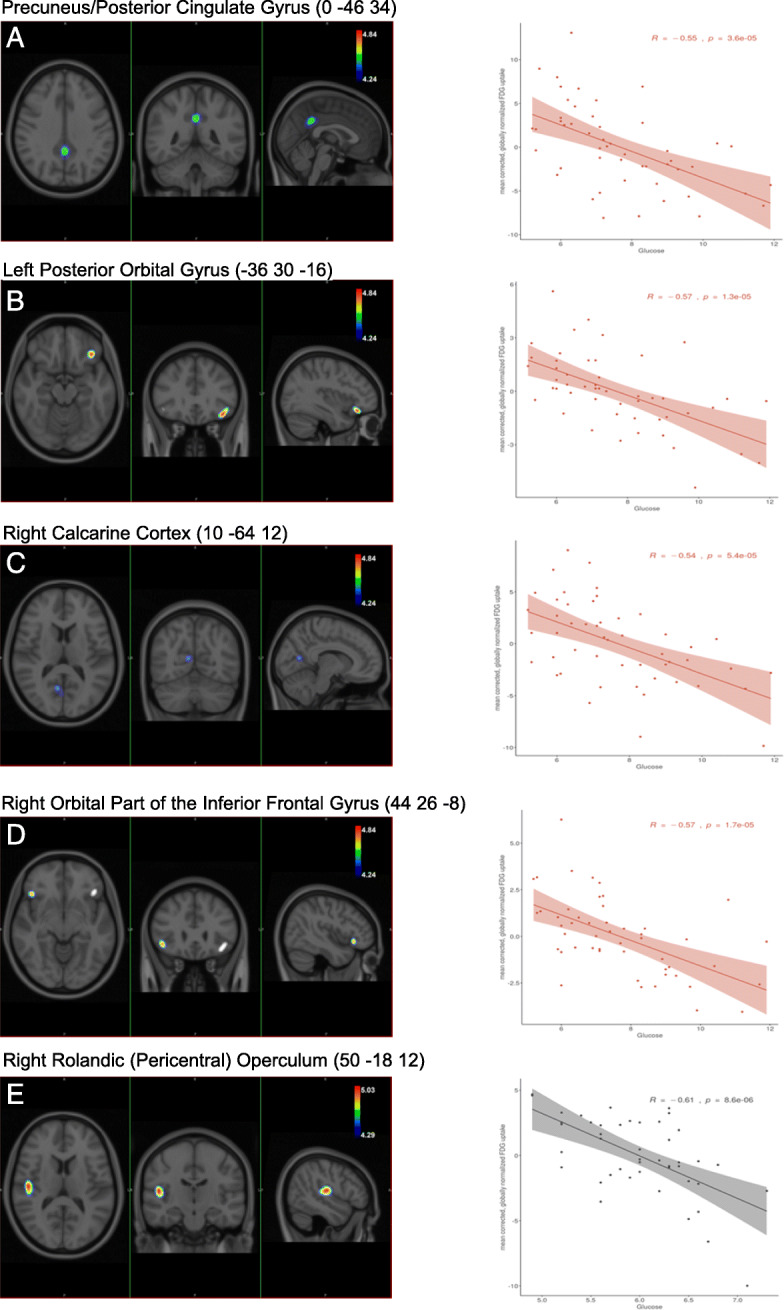
SPM maps showing the results of correlation analyses in both groups. In the diabetes group, the following brain regions show significant correlation with the pre-PET glucose level. **a** Precuneus/posterior cingulate gyrus. **b** Left posterior orbital gyrus. **c** Right calcarine cortex. **d** Right orbital part of the inferior frontal gyrus. **e** In the obese group, the right central operculum (right rolandic pericentral operculum). SPM maps were thresholded at FWE-corrected *P*<0.05 at the cluster level using *k*=40 spatial extend threshold. The colour scales demonstrate the *t* values above the applied *P*_FWE_<0.05 statistical threshold

Further analysis of glucose-sensitive regional metabolic differences was performed in the two study groups separately. In the type 2 diabetic group, the following brain regions were detected to show glucose-sensitive hypometabolism (demonstrated in Fig. 3):

- Precuneus/posterior cingulate gyrus
- Left posterior orbital gyrus
- Right calcarine cortex
- Right orbital part of the inferior frontal gyrus

In the obese group, unlike the diabetic population, we found glucose-dependent reduced metabolism in only one area, namely, the region of the right rolandic (pericentral) operculum as seen in Fig. 3 (*P*_FWE_<0.05). The results of SPM analysis and the parameters of the brain regions are detailed in Table 6.

**Table 6:** Results of statistical parametric mapping (SPM) analyses

	Region	Peak					Cluster	
		***x***	***y***	***z***	***T***_**value**_	***p***_**FWE**_	Volume ^**(cm3)**^	***p***_**FWE**_
**Group difference**	**Precuneus**	−14	−72	32	5.22	0.001	35.7	<0.001
	**Right superior frontal gyrus**	24	42	34	5.2	0.001	4.2	0.008
**Diabetes correlation**	**Left posterior orbital gyrus**	−36	30	−16	4.84	0.010	1.6	0.018
	**Right orbital part of the inferior frontal gyrus**	44	26	−8	4.75	0.013	0.7	0.028
	**Precuneus/ posterior cingulate gyrus**	0	−46	34	4.55	0.022	2.6	0.013
	**Right calcarine cortex**	10	−64	12	4.43	0.031	0.7	0.028
**Obesity correlation**	**Right rolandic (pericentral) operculum**	50	−18	12	5.03	0.007	3.1	0.009

*FWE* family-wise error; *SPM* statistical parametric mapping

Regional brain metabolism did not correlate with the other investigated laboratory parameters.

## Discussion

As MetS impose a significant financial and economic burden on societies, the need for the reduction of its prevalence becomes relevant (Saklayen, 2018; Etchegoyen et al., 2018). Since rising evidence suggests that MetS and its components are associated with the development of brain abnormalities, there is a growing demand for imaging modalities that are sensitive to brain changes developed on the basis of metabolic diseases (Yates et al., 2012).

Previous studies examined the connection between brain metabolism and MetS and its individual components (Yates et al., 2012; Li et al., 2016). A [^18^F]FDG PET study conducted by Willette et al. with 150 cognitively normal, late-middle aged adults involved, revealed lower cerebral regional glucose metabolism in ventral prefrontal, cingulate, temporal, insular, posteromedial cortices and in the cerebellum in association with Higher Homeostatic Model Assessment of Insulin Resistance (HOMA-IR), a parameter assessing metabolic disturbances (Willette et al., 2015). The relationship seemed to be the strongest in the following brain regions: hippocampus, left medial temporal lobe, rostral and posterior cingulate, precuneus and cuneus. Although T2DM is characterised by IR, our study could not fully strengthen the results of the detailed study which might possibly be due to the fact that we did not determine the severity of IR, nor we considered the impact of IR on brain glucose metabolism. Further, participants involved by Willette and co-authors were not taking drugs for glycaemic control at the time of the study or previously, whilst individuals in our study were under antidiabetic medical treatment. Finally, in the mentioned study, only 7 patients had diagnosed T2DM. These might also explain the incoherency between their and our results. In another FDG PET study, Wei Li and co-authors stated that patients with T2DM expressed lower brain glucose metabolism than the non-diabetic individuals (Li et al., 2016). Our result of the comparison analysis could not be actually compared to that of Wei Li et al. regarding that the effects of antidiabetic treatment were not taken into consideration. Additionally, regional brain metabolic alterations were not assessed in the mentioned research. Further, recent research detected reduced frontal metabolism in type 2 diabetics (Baker et al., 2011). In addition, frontotemporal brain regions were registered to show decreased brain glucose uptake in type 2 diabetic individuals, even after controlling for different vascular risk factors (García-Casares et al., 2014). Whilst some studies indicated higher fasting cerebral metabolism in obese patients compared to lean people, others pointed out no associations between obesity and brain glucose uptake (Wang et al., 2002; Iozzo & Guzzardi, 2019). The underlying mechanism behind cerebral metabolic changes related to metabolic diseases is not exactly known. Decreased neuronal function owing to mitochondrial damage, enhanced oxidative stress, disturbances regarding lipid metabolism and neuroinflammation may underpin the association between them (Willette et al., 2015; Craft et al., 2012).

Although several studies supported the association between obesity/T2DM and altered brain glucose metabolism, to our knowledge there is no study so far that have compared the metabolism of the two metabolic diseases.

NeuroQ application based regional metabolic analysis showed no significant difference from normal database which could be explained by the fact that patients involved were well treated and had controlled glycaemic status. However, the same shift trend from normal in both groups may reveal that metabolic diseases potentially have similar pathophysiological effects on brain metabolism. Some brain areas were depicted with lower activity, but this was statistically not significant either. In an FDG PET study, García et al. found reduced frontotemporal metabolism in T2DM compared to healthy cohort (García-Casares et al., 2014). The fact that vascular risk factors are not taken into account in NeuroQ analysis may possibly explain the differences between the results of the current study and that of García and co-workers.

In our research, voxel-bases group comparison revealed hypometabolism in the region of the precuneus in the diabetic group compared to the obese. This could be in line with the results of Apostolova and co-authors who pointed out that elevated blood glucose level (98.4±15.8 mg/dl/5.5±0.88 mmol/L), even in the normal range (reference range 59–149 mg/dl/3.3-8.34 mmol/L) is associated with a decrease regarding [^18^F]FDG uptake in the posterior cortex (Apostolova et al., 2018). Increasing plasma glucose levels causing reduced brain glucose metabolism in the region of the precuneus and the posterior cingulate gyrus detected by Ishibashi K and co-workers could also be in coherence with our result (Ishibashi et al., 2016). Another PET study also supported that glucose loading prior to PET examination induced a reduction in FDG uptake in the precuneus (Ishibashi et al., 2015). In that study, 9 healthy young volunteers (112±22 mg/dl/6.27±1.23 mmol/L) without IR underwent both [^18^F]FDG and O15-H_2_O PET/CT examinations. Besides the precuneus and the posterior cingulate, lateral parietal and frontal cortex also showed decreased metabolism after glucose loading. Beyond the region of the precuneus, we only detected hypometabolism in the rSFG in T2DM. The inconsistency in the results may be because of the difference in the population involved. It should also be noted that median serum glucose level of type 2 diabetics was mildly increased in our study group and they were not in a glucose-loading condition. Further, Baker et al. pointed out decreased metabolism in the posterior cingulate region both in prediabetic and manifest type two diabetic patients (Baker et al., 2011). Finally, Robert Ro. and co-authors examined patients with or without diabetes and found reduced brain glucose metabolism amongst others in the posterior cingulate gyrus (Roberts et al., 2014).

However, when taking pre-PET glucose level into consideration, precuneus did not show hypometabolic difference between the two examined groups. Additionally, the level of pre-PET glucose level seemed to be inversely related to the metabolism of the precuneus. Thus, we assume that hypometabolism in the region of the precuneus is a glucose-dependent regional metabolic alteration, indicating that actual serum blood glucose level influences its FDG uptake. Further, we hypothesise that the extent of glucose hypometabolism in this brain region is rather determined by the actual metabolic state of patients than by diabetes or obesity themselves. Searching for the future clinical significance of our result, we presume that healthy people with glucose levels above the reference value even without metabolic disturbances may have an increased risk for decreasing brain glucose metabolism. Thus, our hypothesis may emphasise the importance of glucose level management, perhaps even outside diabetes and obesity.

The rSFG was also detected to be a hypometabolic brain region in the type 2 diabetic individuals compared to the obese participants. Based on the scope of the existing literature, no previous studies have demonstrated similar results so far. Although the underlying mechanism behind this is not known, we suppose that glucotoxicity may have a role in the appearance of this metabolic change. Besides competing with [^18^F]FDG, excessive amount of glucose may cause neuronal injury and apoptosis of neurons. Thereby, the number of functioning cells decreases, which consequently means a reduction in metabolism as well. We also assume that this brain region could be more vulnerable to diabetes-associated brain changes than other areas.

When examining the groups separately, the subsequent four regions in the diabetic group demonstrated a negative correlation with pre-PET glucose level: precuneus/posterior cingulate cortex, right calcarine cortex, right orbital part of the inferior frontal gyrus and the left posterior orbital gyrus, whilst in the obese group the right rolandic (pericentral) operculum showed reduced metabolic activity. Based on the scope of the available literature data, no previous research has demonstrated pre-PET glucose dependent brain metabolic changes in T2DM and obesity. We presume that these brain regions might be affected first by the metabolic diseases and could be the most vulnerable to the diabetes/obesity-related brain effects. We suppose that parallel with the progression of the diseases, other brain regions will exhibit decreased metabolism. The fact that in the two groups not the same brain regions were detected to show pre-PET glucose dependent metabolic reduction may suggest that obesity and diabetes affect the metabolic response of the different brain areas to varying degrees. Hypometabolism experienced in T2DM in the right calcarine cortex could be interpreted within the framework of the findings of Ying Chui et al., who in a previous fMRI study found co-existing functional intensity and coherence abnormalities in type 2 diabetic patients in the calcarine cortex (Cui et al., 2014). This finding could be associated with long-term diabetes-induced visual impairment and sensory abnormality, that could possibly explain dysmetabolism in the right calcarine cortex (Cui et al., 2014; Liu et al., 2011; Luo et al., 2012). According to the findings of Cyrus A. Raji et al., higher fasting plasma insulin levels, that may be present in DM, are associated with lower volumes in the orbital frontal cortex, possibly leading to hypometabolism in that brain region (Raji et al., 2009). Additionally, although the effects of different antidiabetic drugs on regional brain metabolism were not taken into account during analysis, based on literature data, regular use of metformin could be in connection with lower metabolism in the ventromedial prefrontal cortex, including the orbital gyrus (Huang et al., 2014). The findings of Erin G. and co-authors according to which patients with MetS were reported to have decreased brain activation during pleasantness evaluation of taste stimuli in brain regions connected to the sensory processing of taste information as well as reward value, could potentially provide a reasonable theoretical framework for the interpretation of pre-PET glucose-dependent hypometabolism exhibited in the right rolandic pericentral operculum in the obese group (Green et al., 1620). Regions found in the latter-mentioned study overlap with the central opercular region. Since obesity shares common characteristic features with MetS, we speculate that obesity-induced altered taste processing, similar to that of experienced in MetS, could be associated with reduced metabolism depicted in the opercular region. Further, besides the above-detailed possible mechanisms and the existence of metabolic diseases, other factors such as current metabolic state and individual characteristics may also have a role in the appearance of these region-specific brain metabolic alterations.

Considering the differences between the two applied semi-quantitative evaluations, we can conclude that the voxel-based SPM analysis is more sensitive to detecting subtle changes, whilst NeuroQ using predefined regions is a more robust method suitable to reveal tendencies.

Since other metabolic parameters were not associated with decreased brain metabolism, we suppose that the pre-PET glucose level may be a sensitive marker for the prediction of MetS-associated brain metabolic impairments.

There are important limitations to our study worth noting. First, we had a relatively small sample size that limited our capability to evaluate further correlations between [^18^F]FDG brain metabolism and other measured laboratory parameters. Future research should involve more patients and include follow-up with the aim of investigating how [^18^F]FDG brain metabolism changes over time. Second, we involved controlled diabetic patients under different types of medications (antidiabetics with different mechanism of action, antihypertensive and lipid-lowering drugs and antidiuretics). Third, we did not examine the effect of gender on glycaemic control. Finally, the comparison to healthy control subjects was based on the data base of NeuroQ rather than our own group with matching demographic parameters.

## Conclusion

Pre-PET glucose level dependent hypometabolism was detected in the precuneus in type 2 diabetic individuals compared to non-DM obese participants. To our knowledge, this is the first study to perform pre-PET glucose level corrected comparative analysis of brain metabolism in T2DM and obesity. Pre-PET glucose sensitive brain regions were also revealed in the two study groups separately. Our findings highlight the importance of future correlative studies in the search for mechanistic explanations of the effect of dysmetabolic states on brain glucose metabolism. Since individuals with T2DM/obesity face impairments in daily living activities, imposing a considerable burden of care on both society and family, sensitive diagnostic methods showing alterations in cerebral metabolism have clinical significance as biological markers derived from brain imaging may be effective early indicators of the appearance of cerebrovascular diseases.

## Data Availability

The dataset used and/or analysed during the current study are available from the corresponding author on reasonable request.

## Abbreviations

[^18^F]FDG: 2-[^18^F]-Fluoro-2-deoxy-D-glucose
T2DM: Type 2 diabetes mellitus
DM: Diabetes mellitus
PET/CT: Hybrid positron emission tomography and X-ray computer tomography
pre-PET: Prior to positron emission tomography
MNI: Montreal Neuroscience Institute
SPM: Statistical parametric mapping
FWE: Family-wise error
rSFG: Right superior frontal gyrus
MetS: Metabolic syndrome
WHO: World Health Organisation
MR: Magnetic resonance
PET: Positron emission tomography
BMI: Body-mass index
fMRI: Functional magnetic resonance imaging
MBq: Megabecquerel
Bw: Body weight
CT: Computed tomography
FOV: Field of view
kVp: Kilovolt peak
mAs: Milliamper secundum
HbA1c: Glycated haemoglobin
FDA: Food and Drug Administration (USA)
DICOM: Digital Imaging and Communications in Medicine
ROI: Region of interest
SD: Standard deviation
ANTS: Advanced normalisation tools
FLIRT: FMRIB’s linear image registration tool
FNIRT: FMRIB’s nonlinear image registration tool
FSL: FMRIB Software Library
FWHM: Full width at half maximum
V: Visual cortex
lAVC: Left associative visual cortex
lPVC: Left primary visual cortex
rPVC: Right primary visual cortex
rCbm: Right cerebellum
HOMA-IR: Higher Homeostatic Model Assessment of Insulin Resistance
IR: Insulin resistance
[^15^O]H_2_O: ^15^O dihidrogen oxide
